# Skill mix change between general practitioners, nurse practitioners, physician assistants and nurses in primary healthcare for older people: a qualitative study

**DOI:** 10.1186/s12875-018-0746-1

**Published:** 2018-05-02

**Authors:** Marleen H. Lovink, Anneke J. A. H. van Vught, Anke Persoon, Lisette Schoonhoven, Raymond T. C. M. Koopmans, Miranda G. H. Laurant

**Affiliations:** 1 Radboud Institute for Health Sciences, Scientific Center for Quality of Healthcare (IQ healthcare), Radboud university medical center, P.O. box 9101, 114 6500 HB Nijmegen, The Netherlands; 2 Faculty of Health and Social Studies, HAN University of Applied Sciences, P.O. box 6960, 6503 GL Nijmegen, The Netherlands; 3 Radboud Institute for Health Sciences, Department of Primary and Community Care, Radboud university medical center, P.O. box 9101, 119 6500 HB Nijmegen, The Netherlands; 4 Faculty of Health Sciences, University of Southampton, Tremona Road, Southampton, SO16 6YD UK; 5 Joachim en Anna, Center for Specialized Geriatric Care, Nijmegen, The Netherlands

**Keywords:** Primary healthcare, Older people, Nurse practitioner, Physician assistant, Qualitative research, Skill mix change

## Abstract

**Background:**

More and more older adults desire to and are enabled to grow old in their own home, regardless of their physical and mental capabilities. This change, together with the growing number of older adults, increases the demand for general practitioners (GPs). However, care for older people lacks prestige among medical students and few medical students are interested in a career in care for older people. Innovative solutions are needed to reduce the demand for GPs, to guarantee quality of healthcare and to contain costs. A solution might be found in skill mix change by introducing nurse practitioners (NPs), physician assistants (PAs) or registered nurses (RNs). The aim of this study was to describe how skill mix change is organised in daily practice, what influences it and what the effects are of introducing NPs, PAs or RNs into primary healthcare for older people.

**Methods:**

In total, 34 care providers working in primary healthcare in the Netherlands were interviewed: GPs (*n* = 9), NPs (*n* = 10), PAs (*n* = 5) and RNs (*n = 10*). Five focus groups and 14 individual interviews were conducted. Analysis consisted of open coding, creating categories and abstraction.

**Results:**

In most cases, healthcare for older people was only a small part of the tasks of NPs, PAs and RNs; they did not solely focus on older people. The tasks they performed and their responsibilities in healthcare for older people differed between, as well as within, professions. Although the interviewees debated the usefulness of proactive structural screening on frailty in the older population, when implemented, it was also unclear who should perform the geriatric assessment. Interviewees considered NPs, PAs and RNs an added value, and it was stated that the role of the GP changed with the introduction of NPs, PAs or RNs.

**Conclusions:**

The roles and responsibilities of NPs, PAs and RNs for the care of older people living at home are still not established. Nonetheless, these examples show the potential of these professionals. The establishment of a clear vision on primary healthcare for older people, including the organisation of proactive healthcare, is necessary to optimise the impact of skill mix change.

**Electronic supplementary material:**

The online version of this article (10.1186/s12875-018-0746-1) contains supplementary material, which is available to authorized users.

## Background

‘Ageing in place’ is a way to provide patient-centred healthcare and has been recently introduced in many developed countries. It means that older adults are enabled to grow old in their own home or at least in their community regardless of their physical and mental capabilities [1]. For developed countries, this means a reform that shifts care from hospitals and long-term care facilities to the community [2]. This reform, together with the growing number of older adults, increases the demand on primary healthcare to provide suitable care to the older adults in the community [1]. More and more general practitioners (GPs) are needed and they face a high number of older patients for whom the traditional reactive care delivery system appears unsuitable because they need more pro-active support to live a relative healthy live despite the problems they experience due to ageing. [1, 3]. Furthermore, care for older people lacks prestige among medical students [4]. In the Netherlands, only 0,5% of medical students prefer to proceed a career in care for older people [5], while GP is the most favorite specialization amongst students. However, Zwijsen et al. (2016) showed that GPs struggle to provide care to complex older patients, for example due to insufficient time or insufficient knowledge [6]. Innovative solutions are needed to reduce the workload of GPs, to guarantee the quality of primary healthcare for older people and to contain costs. A solution might be found in skill mix change by introducing nurse practitioners (NPs), physician assistants (PAs) or registered nurses (RNs) into this field.

For the care for older people living at home, NPs, PAs and RNs may work as physician substitutes by independently providing the same services with similar responsibilities as physicians or performing tasks where the physician remains responsible for the tasks performed (referred to as task delegation) [7, 8]. Physician substitution in primary healthcare appeared to achieve at least as good patient outcomes and process of care outcomes as care provided by physicians [9].

NPs, PAs and RNs may also work as physician supplements by providing additional services that complement or extend those provided by the physician [7, 8]. The most commonly performed supplemental tasks by NPs, PAs or RNs in the care for older people living at home are providing proactive healthcare by geriatric assessment, preventive home visiting and/or case management. However, the effects of proactive healthcare for older people vary strongly across studies [10–18]. These mixed results might be related to the different goals and designs of proactive healthcare and also to the organisation of this skill mix change.

As far as we know, studies describing the different forms and characteristics of skill mix change in primary healthcare specifically for older people are scarce and are not described in much detail [7]. No study described the care provider perspective of skill mix change. For successful changes in skill mix, it is also crucial to get insight in the perspective of the professionals involved. Care providers know the daily work very well and have experience with what works and what does not work in organising primary healthcare for older people. Therefore, the aim of this study was to describe the care provider perspective on how skill mix change for older people is organised, what influences it and what the self-perceived effects are of the introduction of NPs, PAs and RNs into primary healthcare.

## Methods

A qualitative approach using focus group interviews and individual interviews to collect data was used. We chose to conduct focus groups as these group interviews provide more information than the sum of individual interviews because of the interaction process [19].

### Setting and interviewees

This study was conducted in primary healthcare in the Netherlands, including both general practice care and community care. In the Netherlands, general practitioners (GPs) are the gatekeepers of healthcare. In 1999, practice nurses (in Dutch: ‘praktijkondersteuners’) were introduced in general practices. These practice nurses support the GP in taking care of patients with chronic diseases according to an evidence-based protocol. NPs and PAs were introduced in general practices in 2001. At the moment, there are more than 3000 practice nurses (some without a nursing background, but most RN) and approximately 140 NPs and 60 PAs working in general practices [20]. In addition, approximately 8800 baccalaureate-educated RNs, called district nurses, work in the community [21]. An unknown, but limited, number of NPs also work in the community. Each care provider in the Netherlands has its own professional profile. Since 2012, NPs and PAs are allowed to perform certain tasks related to diagnosis and treatment, such as independently prescribing drugs [20]. First on an experimental basis, but in 2018 this will be incorporated in the legislation [22]. GPs (working/have been working with an NP or PA), NPs, PAs and RNs working in primary healthcare were recruited for this study. We applied convenience sampling, although it was our goal to purposefully select the participants. Possible participants were contacted by their professional association/network and asked to fill out a short self-developed questionnaire. GPs were also contacted through NPs or PAs who filled out the questionnaire. It was planned to use the questionnaires to apply maximum variation sampling (on age, sex, workplace, years of experience, type of skill mix change) within the homogenous group of each profession. However, we received too little questionnaires to apply maximum variation sampling. Subsequently, everyone who filled out a questionnaire was invited for a (focus group) interview.

### Data collection

Five focus groups and 14 individual interviews were conducted in two rounds. Attendance at each focus group ranged from two to six care providers. First, mono disciplinary focus groups (*n* = 4) were organised as it is known that interviewees feel more comfortable, respected and free to give their opinion without being judged if they perceive that they are alike in some ways [19]. As we were not able to arrange a focus group with at least 4 to 6 GPs within the research period, we decided to conduct individual interviews with GPs. In the first round of interviews, the following topics were discussed: tasks of the different care providers, barriers and facilitators and improvements and effects related to skill mix change. The topics were based on a previous literature study [9] and finalized through discussion among the researchers. Second, a multidisciplinary focus group (*n* = 1) and additional individual interviews were organised in which the results of the first round of interviews were discussed. The multidisciplinary focus group was organised to gain more in-depth information from a multidisciplinary perspective. This gave us the opportunity to confront the different disciplines with differences in views on the topics discussed. Care providers who participated in an individual interview were those interested in participating in the focus group interview, but who could not participate due to busy schedules during the research period. For interview guides, see Additional file 1. The focus group discussions lasted approximately 120 min and were moderated and observed by two or three researchers (MLo, AP, AvV, BJ). MLo was the primary researcher. She attended all focus group interviews and attended a two-day course on how to conduct focus group interviews prior to the event. The observer paid special attention to the interaction and non-verbal communication and made field notes. She also asked additional questions if needed. The individual interviews were conducted face-to-face or by phone by MLo and lasted about 30 min. Data were collected from October 2014 to May 2015.

### Data analysis

All interviews were audio taped and transcribed verbatim. The computer program ATLAS.ti was used to code the interviews by two independent researchers (MLo, AvV) using content analysis. Analysis consisted of open coding, creating categories and abstraction [23, 24]. The researchers (MLo, AvV) met regularly to compare and discuss their codes. The emerging main and subcategories were discussed within the research team (MLo, AvV, MLa, AP). Data saturation was reached in our sample. At the end of each (monodisciplinary focus group) interview we asked participants whether all topics were discussed. The interviews continued until all relevant topics were covered. In the second round of (multidisciplinary focus group) interviews, the first results were confirmed and discussed in-depth until no new information was gathered. However, as it was a convenience sample it is unknown whether another group of participants might provide new information.

## Results

In total, 34 care providers were interviewed: GPs (*n* = 9), NPs (*n* = 10), PAs (*n* = 5) and RNs (n = 10) (see Table 1). Some RNs were specialised in geriatrics. The RNs had European Qualification Framework (EQF) level 4, 5 or 6 [25]. GPs, NPs and PAs had EQF 7.

**Table 1:** Interviewees’ characteristics

Type of interview	Participants	Age Median (IQR)^a^	Sex Female (n)
First round			
Focus group	NP^b^ *gp*^c^ (*n* = 5) NP *gp* and *c*^d^ (*n* = 1)	51.5 (35.3–52)	6
Focus group	PA^e^ *gp* (*n* = 3)	41 (40.5–44.5)	3
Focus group	Practice nurse (n = 3) Practice/district nurse specialized in geriatrics (n = 1)	57 (55–59)	4
Focus group	District nurse (n = 2)	25 and 51	2
Individual	GPs^f^ (*n* = 7)	58 (53.5–59)	2
Second round			
Focus group	NP *c* (n = 1) Practice nurse (n = 1) District nurse (n = 1) District nurse specialized in geriatrics (n = 2)	46 (42–51)	5
Individual	GPs (n = 2) NPs *gp* (n = 3) PAs *gp* (*n* = 2)	45 (36.5–46.3)	6

^a^*IQR* interquartile range

^b^*NP* nurse practitioner

^c^*gp* general practice

^d^*c* community

^e^*PA* physician assistant

^f^GP: general practitioner

The following four main categories were identified: a) roles and tasks of NPs, PAs and RNs, b) responsibilities of NPs, PAs and RNs, c) factors influencing skill mix change and d) impact of skill mix change. After the main categories, accompanying subcategories will be discussed.

### Roles and tasks of NPs, PAs and RNs

NPs and RNs worked at general practices and/or in the community. PAs worked in general practices (see Table 1).

#### NPs, PAs and RNs in general practices

The NPs and PAs performed general consultations for patients from all ages at the general practice, and some NPs and PAs made home visits as well when patients were unable to come to the general practice for a consultation. Most NPs/PAs did not treat all patients, but depending on their experience and practice agreements, they excluded some specific complaints (e.g, stomachache, cardiovascular problems and neurological problems). Most NPs had a more outlined package of tasks than PAs. RNs performed protocol led tasks for the treatment of patients with chronic diseases, such as diabetes mellitus, chronic obstructive pulmonary disease and heart failure. With regard to older people, the RNs, NPs and some PAs delivered proactive healthcare. This proactive healthcare varied from an unplanned preventive home visit, to structural screening on frailty by means of a (partial) geriatric assessment and the organisation of multidisciplinary meetings. During multidisciplinary meetings, the care plan to support the older adult was discussed by the NP, PA or RN and other care providers, including the elderly care physician (i.e. nursing home physician specialist employed by a nursing home organisation) [26]. In some cases, the GP also joined the multidisciplinary meetings depending on the complexity of the case. Tasks of NPs and PAs in healthcare for older people were also medically orientated, they: performed medically screening, diagnosed and prescribed medication, while the RNs focused on nursing care. For examples of tasks see Table 2.

**Table 2:** Tasks in general practices

Provider	Population	Type of care	Examples of tasks^a^
NP^b^, PA^c^	All ages	Medical care^d^	Diagnosis • medical anamnesis (including psychosocial and functional status) • physical examination: examination of hearts, lungs, abdomen, examination of musculoskeletal system, neurological examination, dermatoscopy, ordering blood or feces tests
			Treatment • prescription of medication • small surgical procedures • psychosocial support • referral to other discipline • multidisciplinary treatment and/or support
	Older people	Proactive healthcare (combining medical and nursing care)	varied from an unplanned preventive home visit to structural screening on frailty
RN^e^	All ages	Providing nursing care to patients with chronic conditions such as: • diabetes mellitus • chronic obstructive • pulmonary disease • heart failure	• nursing anamnesis • nursing procedures e.g.: wound care, removing stitches (in order of GP^f^) • psychosocial support • health education • health monitoring • care coordination
	Older people	Proactive healthcare (nursing care)	varied from an unplanned preventive home visit to structural screening on frailty

^a^Tasks were described in the sampling questionnaire and in the (focus group) interviews

^b^*NP* nurse practitioner

^c^*PA* physician assistant

^d^Delineation varied per profession (NPs had a more outlined package of tasks than Pas) and per individual; some NPs/PAs excluded some specific complaints (e.g, stomachache, cardiovascular problems and neurological problems)

^e^*RN* registered nurse

^f^*GP* general practitioner

*‘I have set up the module healthcare for older people together with the GP who did the overarching things, but we [the NPs] shaped the module, which means screening, making agreements on how to screen, who will screen and why … the GPs do not have time for that. I arrange all multidisciplinary meetings and I do all home visits’.* (NP 1.2).

#### NPs and RNs in the community

Two NPs and several RNs (the district nurses) worked outside the general practice. One NP who had a dual employment contract both at the general practice and at a home care organisation performed structural screening on frailty. Another NP employed by an organisation that delivered transmural care developed activities with a specific focus on older people: proactive healthcare, liaison service for GPs and district nurses and developing care paths. The NPs stated that they only performed nursing tasks. They were searching for their role in the care for older people living at home to optimise their scope of practice to education level and legislation. The lack of a vision in organisations on the NPs’ role was perceived as a barrier. The district nurses mainly provided nursing care and proactive healthcare (i.e., networking with other care providers or structural screening of older people similar to the screening performed by providers in general practice and the NPs in the community). The RNs stated that they increasingly worked in collaboration with GPs to provide integrated care for older people. For examples of tasks see Table 3.

**Table 3:** Tasks in the community

Provider	Population	Type of care	Examples of tasks^a^
NP^b^	Older people	Proactive healthcare (nursing care)	• screening of older people • liaison service for GPs^c^ and district nurses • developing care paths
RN^d^	All ages	Nursing care	• support activities of daily living • nursing procedures e.g.: blood pressure control, give an injection (in order of GP) • psychosocial support • health education • health monitoring • care coordination
	Older people	Proactive healthcare (nursing care)	• networking with other care providers structural • screening on frailty

^a^Tasks were described in the sampling questionnaire and in the (focus group) interviews

^b^*NP* nurse practitioner

^c^*GP* general practioner

^d^*RN* registered nurse

### Responsibilities of NPs, PAs and RNs

The RNs worked in close collaboration with the GPs and often they performed delegated tasks, while NPs/PAs worked more independently. Many interviewees reported that the GP had the final responsibility as the patients were listed at the general practice owned by the GP, although several NPs/PAs stated that they had shared responsibility with the GP. Several interviewees said that NPs, PAs and RNs should set boundaries for their responsibilities and were responsible for their own actions. Some stated that these boundaries should be recorded while other stated that it is not possible to do so, because healthcare for older people is too complex.

*‘… that [distribution of responsibilities] I find a hard one. Of course, there are many things that she [NP] does independently, but in principle she always works under our responsibility, but within her own expertise’.* (GP 7.2).

### Factors influencing skill mix change

This category exists of four subcategories: a) coordination, b) collaboration, C) opportunities for NPs, PAs and RNs to provide care to older people alongside GPs and d) acceptability.

#### Coordination

Coordination was deemed to be important in the collaboration in primary healthcare for older people as many different care providers are involved. However, some interviewees reported that different care providers saw each other as competitors. It was perceived to be important not to divide patient care, and most interviewees reported that it would be ideal if the older adult had one central care provider. However, they did not mention which professional this should be. All care providers involved should communicate regularly and preferably use the same (electronic) patient records.

*‘Sometimes when I have a conversation with a patient, I notice that this patient already had screening of which I’m not informed … So there is a lack of communication from the general practice’.* (Nurse with specialty in gerontology and geriatrics from a home care organisation 6.2).

*‘That [coordination] helps a lot. It helps every older adult if you are able to contact experts quickly. I do not need to know everything myself’.* (practice nurse 6.5).

#### Collaboration

Personal characteristics that influenced the collaboration between NPs, PAs or RNs and GPs were as follows: diversity in expertise, type of education, level of experience, personality and affinity with older people. Among GPs specifically, there was a diversity in their willingness to share responsibility for medical patient care with the NP, PA or RN.

*‘Do you as a GP want to share your responsibilities and work with someone else, or do you want to do it on your own? Yes, there are as many opinions as there are GPs’.* (GP 5.3).

Some NPs, PAs and RNs collaborated with one GP while others collaborated with several. Some NPs, PAs and RNs had regular meetings with a GP while others had ad hoc meetings only when a problem needed to be discussed. Many interviewees reported that it was important that the NP, PA and RN could always contact a GP for help when questions regarding the care for the older patient arose. In most cases, the GP was easily accessible.

It was stated that good communication and trust were key factors for a successful collaboration between care providers. The interviewees noticed that the collaboration grows over time, while the NP, PA or RN grows in her function and the GP learns to relinquish patient care.

*‘I think the trust you receive from the GP is a facilitator, the space to act or not to act’.* (PA 2.1).

#### Opportunities for NPs, PAs and RNs to provide care to older people alongside GPs

The interviewees agreed that the complexity of the care for older people living at home provides opportunities for NPs, PAs and RNs to provide care alongside GPs. There was, however, discussion about which professional was most suitable to offer care to older people. The main perceived difference between NPs and PAs was that the NP focuses on nursing and medical procedures and tasks, while the PA focuses mainly on medical procedures and tasks. Some interviewees doubted whether PAs without a nursing background were competent to provide healthcare to older people. In addition, some interviewees wondered whether healthcare for older people was too broad for the scope of practice of NPs. Interviewees saw a role for RNs in primary healthcare for older people as long as it was not complex and worked under supervision of a GP or an NP/PA.

*‘During consultations, it makes no difference whether an NP or PA does it. In healthcare for older people, it can be an added value if you have a nursing background and then, then you still have your nursing part, but if you only do consultations it makes no difference’.* (NP 1.1).

Although the interviewees debated the usefulness of proactive structural screening on frailty, when implemented, it was also unclear who should perform the geriatric assessment. Many interviewees (including the PAs) wondered whether this should be a task of PAs, as they mainly focus on cure, and proactive screening was perceived as care. NPs were reported to be competent to screen older adults with complex care needs. RNs could perform screening of the cases that were expected to be less complex; however, it was not clear whether this should be done by a practice nurse or by a district nurse.

#### Acceptability

Many interviewees reported that patients and their family do not know what to expect from an NP, PA and RN.

*‘Most older patients find it difficult. I always try to explain: it is a new function and I do tasks in the medical domain. I try to explain that as good as possible. In my case I have worked in the practice for a long time and people know me and most accept it. If it needs more explanation then I give that, of course, but most reactions are positive’.* (PA 9.2).

Several NPs, PAs and RNs reported that they experienced problems if they wanted to liaison with a medical specialist at the hospital or refer a patient to the hospital because the medical specialists stated that they only wanted contact with GPs.

According to interviewees, pre-conditions for the implementation of NPs, PAs and RNs were the support of the professional association of GPs and structural financing of primary healthcare for older adults by insurance companies. However, it was stated that this support was not yet optimal.

*‘At the moment, the barriers are the resistance by my GP colleagues. It is often hard to explain to people that within this project [proactive healthcare for older people], in our regional group of general practices we want to employ our NPs. While the regional group of general practices made agreements with the insurance company in which the NP does not fit’.* (GP 5.7).

### Impact of skill mix change

The experienced impact of skill mix change is described under a) added value and b) changing role of the GP, subcategories.

#### Added value

Interviewees considered NPs’, PAs’ and RNs’ contribution to quality of healthcare, provision of patient-centred care and strengthening of the care team in residential homes and homecare organisations to be an added value.

It was perceived that NPs, PAs and RNs contributed to quality of healthcare because, for example, the personal continuity of healthcare was improved as NPs, PAs and RNs were the central care provider for older people. Also, despite the doubts of the (cost) effectiveness of proactive healthcare for older people, it was stated that proactive healthcare provided by NPs, PAs and RNs enables care providers to intervene in a timely manner when something goes wrong.

NPs and RNs characterised themselves above GPs on the nursing domain in knowing their patients very well, having insight into social networks, being easily accessible for patients and family, having a holistic view, working proactively, giving attention to patients, and taking/having time for patients and family.

*‘Patients always say, “It is so funny, if you look at my feet, you always put my socks back on”’.* (NP 1.4).

PAs also stated that they contributed more to patient-centred care because they were easily accessible for patients and family, had an overall view of patients, were well organised, took/had more time for patients and family than GPs. Almost all PAs interviewed had a nursing background and several PAs stated that their added value was due to their nursing background.

*‘The effect is, I think, the background as nurse and practice nurse. That is a background I like because my colleague [GP] says sometimes: [name] you do it a lot more precise than I and that is because you still have a broad view and you still also look, secretly, at the nursing aspects’.* (PA 2.2).

NPs, PAs and RNs were reported to strengthen the care team in residential homes and home care organisation because they: coached, educated and trained them; reminded the care team of their own responsibilities and were easily accessible for the care team.

*‘As a district nurse, I form a link, together with my colleague, between the GP and the care team because the experience was that in the residential home they (GPs) were called too late or too early and, yes, they were very busy with the care for older people’.* (practice nurse 6.4).

#### Changing role of the GP

The introduction of NPs, PAs and RNs changed the role of GPs from a more clinical expert role for all patients to a more coordinating role with focus as clinical expert on the more complex patients. Positive perceived effects were that the workload for the GPs became lower, that their practices could be larger and that they had more time to focus on the more complex patients. Negative perceived effects were that the GPs had less patient contact and less freedom because they should be available for the NP, PA or RN and that the GPs only had consultations for complex patients increasing the caseload as NPs, PAs and RNs only had consultations for less complex patients.

## Discussion

Skill mix change by introducing NPs, PAs and RNs into primary healthcare for older people appeared to be only at the start of its development. In most cases, healthcare for older people was only a small part of the tasks of NPs, PAs and RNs (i.e. they do not solely focus on older people). The tasks they performed and their responsibilities in healthcare for older people differed between as well as within professions and were not always in line with their education and legal authorisations; underuse of competences existed. Full potential of NPs, PAs and RNs in the care for older people living at home was, according to the interviewees, not yet reached, partly because a vision of the role of each professional in primary healthcare for older people was lacking. There was also discussion about how to organise proactive healthcare for older people where these professionals could have a leading role. In addition, skill mix change required team performance, collaboration, trust and acceptance of each other’s expertise instead of competition. Skill mix change also affected the role of the GP and appeared to enhance quality of healthcare.

In accordance with our study, several studies have reported variation in the level of autonomy of NPs (and PAs, and RNs) [27, 28]. In the Netherlands, NPs and PAs are allowed to perform certain tasks related to diagnosis and treatment independently, such as the prescribing of drugs [29]. In contrast to this legislation, interviewees in the current study stated that the GP had final responsibility for patient care. There are three possible explanations for the fact that, in our study, the GP was reported to have final responsibility: 1) GPs, NPs and PAs do not know the legal boundaries of skill mix changes and the competences of NPs and PAs, 2) in the Netherlands, patients are listed at a general practice which is owned by a GP, which might enhance the sense of responsibility of GPs [26] and 3) research has shown that known that in collaborations care providers aim to maintain their power and that conflict or dissatisfaction may occur if their power is challenged [30, 31]. Although power and autonomy are important in collaborations, care providers might respond more positively regarding collaborations if they are based on trust rather than power [31]. In line with our study several studies have reported that the longer a GP works with an NP (or a PA, or a RN), the more (s)he trusts the NP and the more (s)he acknowledged the expertise of this professional [27, 30, 32]. One of these studies showed that trust is positively related to the extent to which roles are accepted, demonstrated competences and good communication [30].

Collaboration in primary healthcare has been studied extensively, and two models of collaboration have been developed [33–35]. The Four-Dimensional Model of Collaboration, which consists of internalisation, shared goals and vision and governance [33, 34], and the Gears Model of Factors Affecting Interprofessional Collaboration, consisting of individual, micro, meso and maso factors [35]. Both models state that collaboration is influenced by factors on different levels: individual, team, information exchange, and governance [33–35]. Next we will give some examples of how these models might be applied to primary healthcare for older people to improve collaboration in the light of skill mix change. For example, in the current study, there was discussion about the uniqueness of each care provider. A prerequisite for collaboration is mutual acquaintanceship (i.e., knowledge of each other’s values and professions). Furthermore, care providers should not see each other as competitors. All care providers that are involved in the care for an older adult should share the same goals and vision to provide the best available care to older people. This process should be facilitated through formalisation by means of digital patient records and by recording the responsibilities of the various care providers involved in the care for an older adult [33–35]. In addition, on a higher level, discussions should be held on how to organise the care for older people living at home, such as who to employ from which setting, whether it is desirable to involve multiple care providers in the care for an older adult, etc. These discussions could be held at the state level, the professional association level, the insurance company level and the level of collaborating general practices [33–35].

This study has some limitations that should be considered while interpreting the results. First, the division of the interviewees in focus groups was not optimal. Some focus groups were very small, which diminished the interaction process between interviewees [19]. In the multidisciplinary focus group, only one NP and different types of RNs participated. Therefore, no interaction with PAs and GPs could occur. To gain insight into their views on the results of the first round, PAs and GPs were interviewed individually. The difficulties in finding interviewees for the focus groups were due to the high workload related to the reforms in primary healthcare in the Netherlands. Second, self-reporting of activities might lead to social desirability bias [36]. Interviewees might have reported their tasks and responsibilities different from reality. To overcome this problem, an observational study on the role of NPs, PAs and RNs in primary healthcare for older people should be carried out. Third, the study focused on the perspective of the providers and therefore lacks the perspective of older people and their family. It is important to explore the experiences and opinions of older people with skill mix change and to determine both their needs and the acceptability of the concept. The results of our current study provide detailed input for interviews regarding skill mix change. Especially, the acceptability and (un)familiarity with NPs, PAs and RNs are important topics to discuss, because it may result in ideas how to improve skill mix change.

## Conclusion

Although NPs, PAs and RNs are involved in the care for older people living at home, a huge variation in tasks and responsibilities between and within professions exists. A clear vision on care for older people, including the organisation of proactive healthcare and roles and responsibilities of team members, is necessary to increase the impact of skill mix change on quality of healthcare. The role of the GP as the traditional care provider needs to change to maintain quality. All team members should be informed about legislation to ensure that NPs, PAs and RNs perform to their full potential.

## Additional file

Additional file 1: Interview guides. (DOCX 16 kb)

## Abbreviations

NP: nurse practitioner
PA: physician assistant
RN: registered nurses
