# Supplementary material for: Skill mix change between general practitioners, nurse practitioners, physician assistants and nurses in primary healthcare for older people: a qualitative study
Source: BMC Fam Pract. 2018 May 2;19:51. doi: 10.1186/s12875-018-0746-1 (PMC5932890; doi:10.1186/s12875-018-0746-1)
Supplement: Supplementary file 1 — Interview guides. (DOCX 16 kb) [file 12875_2018_746_MOESM1_ESM.docx]

**Interview guides**

Interview guide *first* round of (focus group) interviews

**What are your tasks in primary healthcare for older people?**

How does your position/occupation relate to the position/occupation of other professionals?

What is your role in relation to other professionals?

Who performs which tasks?

Would you describe your tasks as substitution, delegation or supplementation?

**What is the effect of skill mix change?**

**What are barriers and facilitators to skill mix change?**

What are chances, challenges, threats, conditions, and boundaries for skill mix change?

**How should skill mix change be organized in the future?**

**What is your role in the future?**

Is it possible that another professional performs your tasks?

Is it possible that you take over tasks from another professional?

What will your position/occupation look like in 5 or 10 years?

Who should perform which tasks?

Interview guide *second* round of (focus group) interviews

Interviewees received beforehand a summary of the findings of the first round of (focus group) interviews.

**Do you recognize the results of the first round of (focus group) interviews?**

**Are the results complete?**

**What is the optimal model of skill mix change (for the patient) in what circumstances?**

Why should skill mix change be organised in this way?

Which professionals work together?

What is the goal of skill mix change?

**Why is the optimal model of skill mix change not yet a reality?**

Topics to discuss:

Tasks

Responsibilities

Effects of skill mix change

Barriers and facilitators to skill mix change
